# In Vivo Efficacy of Voriconazole in a *Galleria mellonella* Model of Invasive Infection Due to Azole-Susceptible or Resistant *Aspergillus fumigatus* Isolates

**DOI:** 10.3390/jof7121012

**Published:** 2021-11-26

**Authors:** Sana Jemel, Jacques Guillot, Kalthoum Kallel, Grégory Jouvion, Elise Brisebard, Eliane Billaud, Vincent Jullien, Françoise Botterel, Eric Dannaoui

**Affiliations:** 1 Dynamic, Ecole Nationale Vétérinaire d’Alfort, Université Paris Est Créteil, 94010 Créteil, France; jemelsana.benayed@gmail.com (S.J.); jacques.guillot@oniris-nantes.fr (J.G.); gregory.jouvion@vet-alfort.fr (G.J.); francoise.botterel@aphp.fr (F.B.); 2 Faculté de Médecine de Tunis, Université Tunis EL Manar, Tunis 1007, Tunisia; kallelkalthoum@gmail.com; 3 UR17SP03, Centre Hospitalo-Universitaire La Rabta, Jabbari, Tunis 1007, Tunisia; 4 Dermatology, Parasitology-Mycology Department, Ecole Nationale Vétérinaire de Nantes, Oniris, 44300 Nantes, France; 5 Unité de Neuropathologie Expérimentale, Institut Pasteur, 75015 Paris, France; elise.brisebard@oniris-nantes.fr; 6 Laboratoire d’Histopathologie, Université de Lyon, VetAgro-Sup, 69280 Marcy l’Etoile, France; 7 Service de Pharmacologie APHP, Hôpital Européen Georges Pompidou, 75015 Paris, France; eliane.billaud@aphp.fr (E.B.); vincent.jullien@aphp.fr (V.J.); 8 Faculté de Médecine, Université de Paris, 75006 Paris, France; 9 Unité de Parasitologie-Mycologie, Service de Microbiologie, APHP, Hôpital Européen Georges Pompidou, 75015 Paris, France

**Keywords:** *Aspergillus*, antifungals, *Galleria mellonella*, voriconazole, azole resistance

## Abstract

*Aspergillus fumigatus* is an environmental filamentous fungus responsible for life-threatening infections in humans and animals. Azoles are the first-line treatment for aspergillosis, but in recent years, the emergence of azole resistance in *A. fumigatus* has changed treatment recommendations. The objective of this study was to evaluate the efficacy of voriconazole (VRZ) in a *Galleria mellonella* model of invasive infection due to azole-susceptible or azole-resistant *A. fumigatus* isolates. We also sought to describe the pharmacokinetics of VRZ in the *G. mellonella* model. *G. mellonella* larvae were infected with conidial suspensions of azole-susceptible and azole-resistant isolates of *A. fumigatus*. Mortality curves were used to calculate the lethal dose. Assessment of the efficacy of VRZ or amphotericin B (AMB) treatment was based on mortality in the lethal model and histopathologic lesions. The pharmacokinetics of VRZ were determined in larval hemolymph. Invasive fungal infection was obtained after conidial inoculation. A dose-dependent reduction in mortality was observed after antifungal treatment with AMB and VRZ. VRZ was more effective at treating larvae inoculated with azole-susceptible *A. fumigatus* isolates than larvae inoculated with azole-resistant isolates. The concentration of VRZ was maximal at the beginning of treatment and gradually decreased in the hemolymph to reach a C_min_ (24 h) between 0.11 and 11.30 mg/L, depending on the dose. In conclusion, *G. mellonella* is a suitable model for testing the efficacy of antifungal agents against *A. fumigatus*.

## 1. Introduction

*Aspergillus fumigatus* is a filamentous fungus ubiquitously found in the environment and is responsible for life-threatening opportunistic infections in humans and animals. The increasing use of immunosuppressive and long-term corticosteroid therapies, and new risk-factors, such as severe influenza or SARS-CoV-2 infections, have resulted in an increase in the frequency of invasive aspergillosis worldwide [1,2,3]. *A. fumigatus* is the most common species causing invasive aspergillosis, followed by *A. flavus*, *A. terreus* and *A. niger* [4]. As clinical signs are often unspecific, diagnosis and therapy of invasive aspergillosis is delayed, leading to a high mortality rate [5]. Azoles are the drugs of choice for the treatment of invasive aspergillosis, and amphotericin B (AMB) is an alternative in case of intolerance [6]. Voriconazole (VRZ) has long been the azole indicated as a first line treatment of invasive aspergillosis [7]. However, the extensive use of azole drugs in the prevention and treatment of fungal infections, and the extensive use of fungicides in agriculture, have contributed to the emergence of azole resistance in *A. fumigatus* [8], making the management of invasive aspergillosis more complex [9]. The resistance of this fungal species to azoles is mainly related to changes in the enzyme target of the drugs involved, which is in the ergosterol biosynthesis pathway, via mutation of its gene [10]. The emergence of resistance makes alternative treatment options for invasive aspergillosis necessary [9,11].

Standardized techniques for assessing the in vitro activity of antifungal drugs are now well known, but the use of in vivo models is still required for the validation of therapeutic options. Recently, several invertebrate animal models have proved interesting for studying the virulence of different fungal species, but also the efficacy of different treatments [12,13]. These new models have many advantages and are also cheaper than mammalian models and do not require the same ethical considerations. Among these invertebrate models, *Galleria mellonella* is widely used and characterized by its ability to survive at temperatures at or above 37 °C, at which certain virulence factors of fungi are expressed. This model has been validated to test the virulence of microorganisms (bacteria, fungi and protozoa) [14,15], but also to evaluate the effectiveness of antimicrobials [16,17].

The aim of this study was to establish and evaluate a model of invasive aspergillosis in *G. mellonella* with azole-susceptible or azole-resistant *A. fumigatus* isolates. Using this model, the antifungal drug responses to AMB and VRZ were evaluated. Furthermore, we evaluated the pharmacokinetics of VRZ in larvae.

## 2. Materials and Methods

### 2.1. Isolates, Medium and Growth Conditions

Three clinical *A. fumigatus* isolates were used in this study. Their identification was confirmed by sequencing part of beta-tubulin gene. The *CYP51A* gene and its promoter were also sequenced. One isolate (HEGP064) had a wild type *CYP51A* sequence, one isolate (HEGP4017) presented a G54W mutation, and one isolate (HEGP2666) had a L98H point mutation in the *CYP51A* gene in combination with a 34-bp tandem repeat in the promoter (TR34/L98H). Fungal isolates were kept at −20 °C in glycerol until use. Subcultures were performed on Sabouraud (VWR, Fontenay-sous-Bois, France) with chloramphenicol (Sigma-Aldrich, Saint Quentin-Fallavier, France). They were incubated for 7 days at 37 °C to obtain sufficient sporulation.

### 2.2. In Vitro Antifungal Susceptibility to Azoles

Antifungal susceptibility was determined by the reference microdilution broth technique following the recommendations of the Antifungal Susceptibility Testing Subcommittee of the European Committee on Antimicrobial Susceptibility Testing (AFST-EUCAST) (16). Minimal inhibitory concentrations (MICs) were determined for VRZ, itraconazole (ITZ), posaconazole (PSZ) and AMB. Isolates were considered resistant when MIC was >1 mg/L for ITZ and VRZ, and when MIC was >0.25 mg/L for PSZ according to the AFST-EUCAST breakpoints.

### 2.3. Galleria mellonella Infection Model

Larvae of *G. mellonella* (Kreca^®^ Ento-Feed BV, Ermelo, The Netherlands) were used throughout the experiments. Larvae were kept in their boxes containing food in the dark at 18 °C before use. All the larvae selected for the experiments had normal mobility with the capacity to turn over on the ventral side and had a uniform color. Only larvae with a weight between 100 and 300 mg were used. In each set of experiments, larvae were randomly distributed.

After culturing of the three *A. fumigatus* isolates, the inoculum was prepared in phosphate-buffered saline containing 0.01% of Tween 20 (PBST). The slope of the culture was washed with PBST, and then the suspension obtained was filtered using a sterile gauze pad to obtain the initial suspension. Spore suspensions were adjusted to the required concentration by counting conidia in a hemocytometer. The different concentrations (10^8^, 3.10^7^, 10^7^, 3.10^6^, 10^6^ and 10^5^ conidia/mL) were obtained by serial dilutions in 0.01% PBST.

For each concentration of *A. fumigatus* conidia, a group of 10 larvae was inoculated. The injection was carried out with 10 µL in the ventral side of the last proleg by using a Hamilton^®^ syringe. After inoculation, larvae were stored in the dark at 37 °C and mortality was evaluated daily for one week. Two control groups were used: larvae in the first were inoculated with PBST, and the second group consisted of untouched larvae. From the mortality data, the LD_90_ (i.e., the inoculum size that gave 90% of mortality) for each of the isolates was calculated. Experiments were performed in duplicate.

### 2.4. Treatment

For treatment experiments, only commercial preparations of antifungals were used. Stock solutions were prepared, aliquoted and then stored at −20 °C until use. For AMB, the powder of Fungizone^®^ (Bristol Myers Squibb) was dissolved in 10 mL of sterile distilled water to obtain a stock solution of 5 mg/mL. To obtain the different dosages (20, 5, and 2.5 mg/kg/day corresponding to 4, 1 and 0.5 µg/larva), dilutions were performed in 5% glucose. For VRZ, the Vfend^®^ (Pfizer, Paris, France) powder was dissolved in 19 mL of 0.9% saline to obtain a stock solution at 10 mg/mL. The different dosages required (40, 20, 10, 5, and 2.5 mg/kg/day corresponding to 8, 4, 2, 1 and 0.5 µg/larva) were obtained by carrying out dilutions in 0.9% saline. The antifungal doses for *G. mellonella* were calculated taking in consideration the therapeutic doses used in humans.

All groups, consisting of 10 larvae, were infected by injection of 10 µL of the corresponding LD_90_ of each *A. fumigatus* isolate. A volume of 10 µL of each antifungal was injected in the haemocoel of larvae with different doses at 2, 24 and 48 h after infection. Following antifungal treatment, larvae were maintained at 37 °C for 7 days. Larvae survival was monitored daily, and larvae were considered dead when they did not respond to stimulation. Two control groups were used, the first group consisted of infected larvae injected with 0.9% saline at 2, 24 and 48 h after infection and the second group (to assess toxicity) was composed of non-infected larvae injectedwith the highest dose of antifungal (4 µg/larva for AMB and 8 µg/larva for VRZ). All experiments were performed in duplicate, and results were pooled.

### 2.5. Direct Examination and Histopathology

To prove fungal infection in the dead larvae, they were ground with an Omni Tissue Master homogenizer. Smears, made from the homogenate, were stained with Grocott’s methenamine silver. The smears were analyzed by optical microscopy.

Histopathology was performed on 24 infected larvae. Those 24 larvae were distributed in two groups of 12 larvae which were inoculated with either the VRZ-susceptible HEGP064 or the VRZ-resistant HEGP2666 isolate, using the corresponding LD_90_. Larvae were treated with various doses of VRZ (0, 2, 4, 8 µg/larva) at 2, 24 and 48 h after infection. Larvae were stored in dark at 37 °C and were sacrificed on day 1, 2 or 3 after treatment. Therefore, one larva was tested for each condition (strain, dose of VRZ and time-point after treatment). Larvae were fixed by injections of formalin 10% in different parts of the larva (total of 30 µL) before being kept at 4 °C in formalin until paraffin embedding. Each larva was cut in two parts longitudinally before being dehydrated and embedded in paraffin. Histopathological sections were performed according to a standard protocol and stained with hematoxylin-eosin and Grocott’s methenamine silver to assess the location of fungal elements. Sections of each larva were analyzed by optical microscopy and classify depending on the extension and type of lesions. The histopathological analysis was carried out in double blind by two experienced examiners (Table S1).

### 2.6. Pharmacokinetics of VRZ in Hemolymph

Pharmacokinetic profiles of VRZ were evaluated in larvae hemolymph. Non-inoculated larvae and larvae infected with LD_90_ of the azole-susceptible isolate (HEGP064) were injected with 10 µL of different concentrations of VRZ (0.5, 1, 2, 4, 8 or 16 µg/larva). The high dose of 16 µg/larva was evaluated in the pharmacokinetic study although it was not tested in survival experiments. Each group was composed of 16 larvae. For infected larvae, VRZ was injected 2 h after inoculation. Larvae were stored in dark at 37 °C after VRZ injection. The hemolymph of 4 larvae of each group was collected at pre-fixed time point after VRZ injection (0.5, 2, 5, 8, 16 or 24 h). Larvae were sacrificed by placing them during 10 min at −20 °C. An incision was made near the last pseudopod with a sterile needle and a light pressure was made to release the hemolymph. Samples from 4 randomly chosen larvae were pooled, weighted, and stored at −20 °C.

VRZ was determined using a previously described liquid chromatography with tandem-mass spectrometry method that was validated for 50 µL plasma samples [18]. As the volume of hemolymph samples could not be measured precisely, the volume of the reagents used for samples’ treatment was adjusted on each sample’s weight, assuming a density of 1, in order to have in each extract the concentration that would have been obtained in a 50 µL sample.

### 2.7. Pharmacokinetic Analysis

Maximum concentration (C_max_) and the lowest quantifiable concentration (C_min_) were directly observed from the data. Area under the curve from time 0 to 24 h (AUC_24_) was determined by the trapezoidal rule in Microsoft Excel, by using for each time-point the mean of measured concentrations.

### 2.8. Statistical Analysis

The mortality curves were generated by Kaplan–Meier method and compared by the log-rank test. For determination of LD_90_ (inoculum concentration that gave a 90% mortality at day 7), the percentage of mortality at day 7 post infection as a function of the log of the inoculums was analyzed. A regression curve, according to a sigmoid dose-response model, was obtained by nonlinear regression, by setting the minimum and maximum mortality values to 0 and 100%, respectively. The correlation coefficient (*R*^2^) was calculated. From the curve regression, the inoculums values corresponding to LD_90_ were determined. All analyzes were performed using GraphPad Prism V.3.0 software for Windows (GraphPad Software, San Diego, CA, USA). A value of *p* < 0.05 was considered to be significant.

## 3. Results

### 3.1. In Vitro Antifungal Susceptibility to Azoles

The in vitro antifungal susceptibility of the *A. fumigatus* isolates as determined by the EUCAST microdilution broth technique is shown in Table 1. Isolate HEGP064 was susceptible to all azoles; HEGP4017 was resistant to ITZ and PSZ but not to VRZ; and the third isolate HEGP 2666 was pan-azole resistant. All the isolates were susceptible to AMB (MIC of 0.5 mg/L).

### 3.2. Galleria mellonella Survival Assay and Determination of LD_90_ and LD_10_

After inoculation with 10^5^ to 10^8^ conidia/mL for each of the three *A. fumigatus* isolates, death was reported daily for 7 days. In the untouched group of larvae and in larvae inoculated with PBST mortality was <10%. In the group of larvae infected with the highest level of inoculum, the mortality rate was ≥90% at day 7 post infection for each of the three isolates. With the same inoculum concentration, the virulences of the three isolates were comparable. A 10^5^ conidia/mL inoculum was associated with 5%, 10% and 15% of mortality at day 7 post-infection for HEGP064, HEGP4017 and HEGP2666, respectively. With the highest level of inoculum of 10^8^ conidia/mL, the mortality rates of larvae at day 7 post infection were 95%, 100% and 90% for the three isolates respectively.

**Figure 1:**
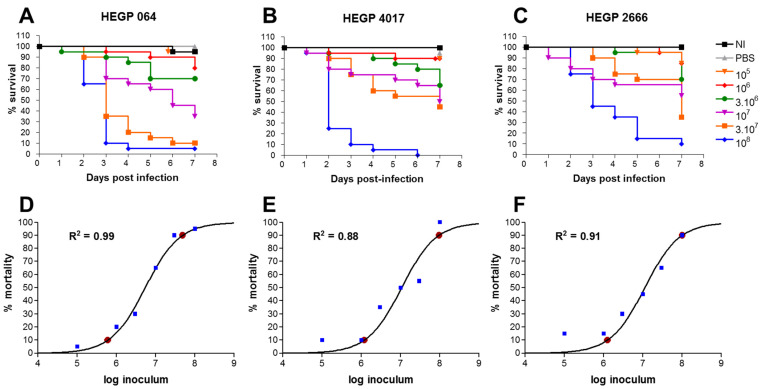
Survival curves of groups of *G. mellonella* larvae inoculated with *A. fumigatus* isolates, HEGP064 (**A**), HEGP4017 (**B**) and HEGP2666 (**C**), at different concentrations from 10^5^ to 10^8^ conidia/mL. Inoculum–mortality relationship obtained by nonlinear regression curves for isolates HEGP064 (**D**), HEGP4017 (**E**) and HEGP2666 (**F**). Experimental points are displayed as blue squares and LD_10_ and LD_90_ as red dots. NI: non-infected larvae, PBS: Phosphate buffered saline, LD: lethal dose. Data from 2 experiments were pooled. Number of larvae = 20 for all isolates and all groups.

There was a clear relationship between the inoculum size and the mortality rate (Figure 1). This is supported by the high *R*^2^ values ranging from 0.88 to 0.99. From the nonlinear regression analysis, LD_90_ values were calculated for the three isolates (Table 2).

### 3.3. Evaluation of Antifungal Efficacy in the Lethal Model of G. mellonella

#### 3.3.1. Evaluation of Amphotericin Efficacy

**Figure 2:**
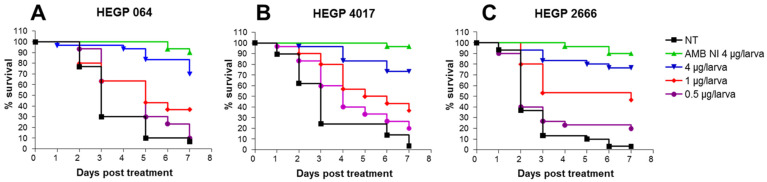
Survival curves of groups of *G. mellonella* larvae inoculated with LD_90_ of *A. fumigatus* isolates HEGP064 (**A**), HEGP4017 (**B**) and HEGP2666 (**C**) and treated with 4, 1 or 0.5 µg/larva of amphotericin B 2, 24 or 48 h after infection. AMB: amphotericin B, NT: not treated larvae. AMB NI 4: non-infected larvae treated with the highest dose of AMB (4 µg/larva). Data from 3 experiments were pooled. Number of larvae = 30 for all isolates and all groups.

For each isolate, larvae infected with LD_90_ were treated with 0.5, 1 or 4 µg amphotericin/larva, 2, 24 or 48 h post infection. For the three isolates, infected larvae treated with AMB showed improved survival compared to untreated controls, and the survival rate was dose dependent. At day 7 post infection, the mortality rates were 95%, 100% and 100% in larvae infected by HEGP064, HEGP4017 and HEGP2666, respectively (Figure 2). In contrast, when larvae were treated with 4 µg of AMB, the mortality rates were only 30%, 25% and 20% with HEGP064, HEGP4017 and HEGP2666, respectively. Treatment with AMB at 4 µg significantly decreased mortality compared to 1 µg in larvae infected with HEGP064 (*p* = 0.003), HEGP4017 (*p* = 0.003) and HEGP2666 (*p* = 0.01). Treatment with 0.5 µg/larva significantly reduced mortality compared to untreated larvae for HEGP064 (*p* = 0.03) and HEGP4017 (*p* = 002) but not for HEGP2666 (*p* = 0.1828).

#### 3.3.2. Evaluation of VRZ Efficacy

**Figure 3:**
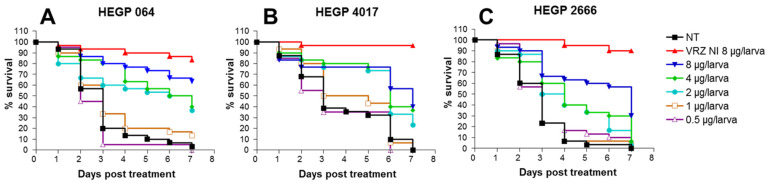
Survival curves of groups of *G. mellonella* larvae inoculated with LD_90_ of *A. fumigatus* isolates HEGP064 (**A**), HEGP4017 (**B**) and HEGP2666 (**C**) and treated with 8, 4, 2, 1 or 0.5 µg/larva of voriconazole 2, 24 or 48 h after infection. VRZ: voriconazole, NT: not treated larvae. VRZ NI 8: non-infected larvae treated with the highest dose of VRZ (8 µg/larva). Data from 2 to 3 experiments were pooled. Number of larvae = 30 for isolates HEGP2666 and HEGP4017 for all groups and 20 for HEGP064 for all groups.

For each isolate, larvae infected with LD_90_ were treated with 0.5, 1, 2 or 4 µg of VRZ/larva, 2, 24, and 48 h post infection. In the untreated group, all larvae were dead in 7 days. Larvae infected with VRZ-susceptible isolates (MIC < 1 mg/L for both HEGP064 and HEGP4017) showed better survival when treated with 2, 4 or 8 µg/larva compared to untreated larvae with a dose-dependent relationship (Figure 3). Mortality was 95% in larvae treated with 0.5 µg/larva and decreased to 80%, 65%, 55% or 35% after treatment with 1, 2, 4 or 8 µg/larva, respectively, among HEGP064-infected larvae.

Despite the fact that treatment with VRZ reduced the mortality of larvae infected with the VRZ-resistant isolate (HEGP2666), this improvement remained less than that observed in larvae infected with VRZ-susceptible isolates. In fact, after treatment with 8 µg/larva, 7-day mortality was only 30% for larvae infected with HEGP064, and it was 70% for larvae infected with HEGP2666. Administration of 4 µg/larva reduced mortality to 60% for the two groups of larvae infected with VRZ-susceptible isolates (HEGP064, HEGP4017), but mortality was 95% for group of larvae infected with the VRZ-resistant isolate (HEGP2666). Only VRZ concentrations of 2 (*p* = 0.0019), 4 (*p* = 0.0013) and 8 µg/larva (*p* < 0.0001) decreased mortality of larvae infected with HEGP2666.

### 3.4. Direct Examination and Histopathological Analysis of Infected G. mellonella Larvae

Direct examination of homogenates after methenamine silver staining showed branching hyphae, demonstrating the development of an invasive *Aspergillus* infection in larvae inoculated with a suspension of conidia (Figure S1).

**Figure 4:**
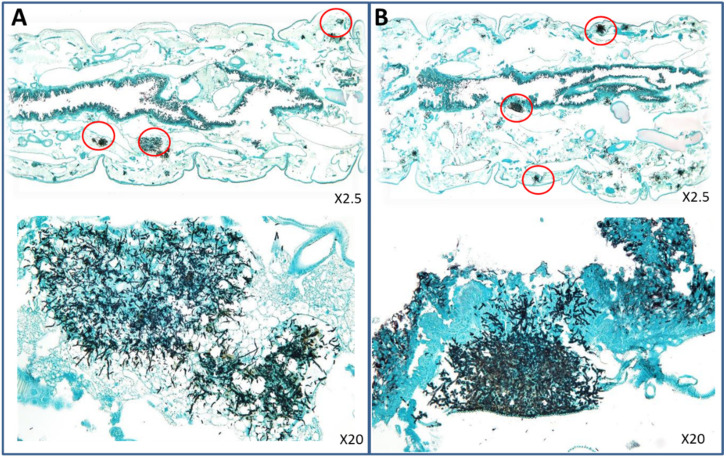
Histopathology of *Galleria mellonella* larvae infected with HEGP064 (**A**) or HEGP2666 (**B**) of *Aspergillus fumigatus* 2 days after infection. Larvae were not treated. Circles indicate tissue invasion. The same type of invasive infection was observed in the two larvae.

**Figure 5:**
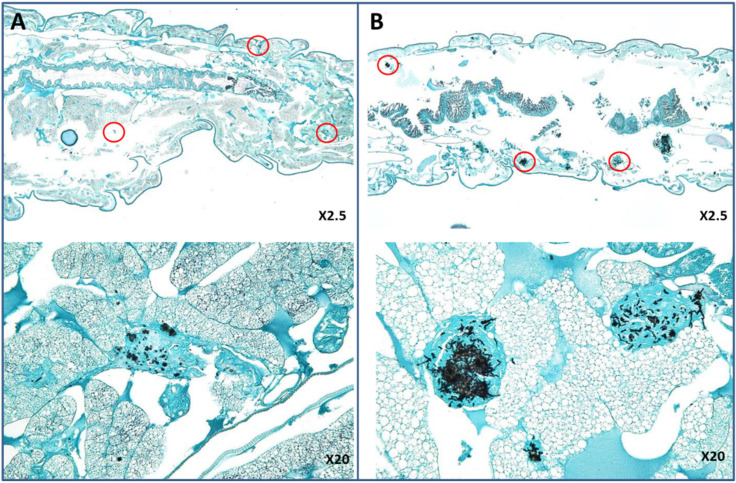
Histopathology of *Galleria mellonella* larvae infected with HEGP064 (**A**) or HEGP2666 (**B**) isolates of *Aspergillus fumigatus* 3 days after infection and treated with voriconazole at 2 µg/larva. Circles indicate tissue invasion. (**A**) Type 1; poorly disseminated infection with presence of conidia in the lesions. (**B**) Type 2b; disseminated infection with predominance of hyphae.

Histopathological analysis revealed the same type of invasive infection in non-treated larvae infected with VRZ-susceptible (HEGP064) or VRZ-resistant (HEGP2666) isolates. The infection was disseminated in different parts of the larvae (Figure 4). The efficacy of VRZ was globally more visible in larvae infected with the VRZ-susceptible isolates compared to those infected with the VRZ-resistant isolate. At day 3, lesions were classified as stage 1 for HEGP064-infected larvae after treatment with 2 or 4 µg/larva of VRZ, whereas it was classified 2b for HEGP2666-infected larva (Figure 5).

### 3.5. Pharmacokinetics of VRZ in Hemolymph

**Figure 6:**
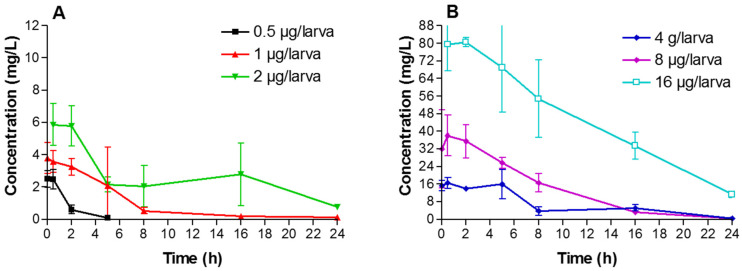
Pharmacokinetic profiles of voriconazole in non-infected larvae following administration of 0.5, 1 or 2 µg/larva (**A**) and 4, 8 or 16 µg/larva (**B**).

**Figure 7:**
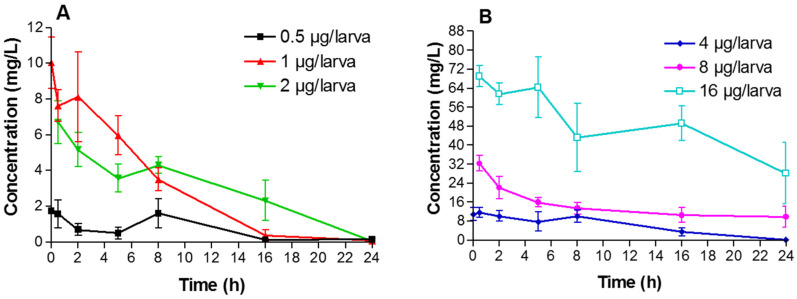
Pharmacokinetic profiles of voriconazole in HEGP064 infected larvae following administration of 0.5, 1 or 2 µg/larva (**A**) and 4, 8 or 16 µg/larva (**B**).

Different VRZ doses (0.5, 1, 2, 4, 8 and 16 µg/larva) were injected to *A. fumigatus*-infected and non-infected larvae. Mean VRZ concentration–time profiles for each dose are shown in Figure 6 and Figure 7.

A peak concentration occurred 30 min after administration of VRZ. Values of AUC_0–24_, C_max_ and C_min(24h)_ were determined for each VRZ dose (Table 3). In non-infected larvae, C_max_ of VRZ ranged from 2.5 to 79.5 mg/L depending on the dose. Concentration decreased gradually in the hemolymph, and the VRZ C_min(24h)_ was 0.1 for the lowest dose (0.5 µg/larva) and 21 mg/L for the highest dose (16 µg/larva). Pharmacokinetics of VRZ were also monitored in infected larvae. C_max_ ranged from 1.7 to 69.0 mg/L and C_min(24h)_ ranged from 0.1 to 28.2 mg/L, depending on the dose. Overall, similar pharmacokinetic parameters were observed in infected larvae compared to non-infected larvae.

## 4. Discussion

We observed a clear relationship between *A. fumigatus* inoculum size (used for infection) and larval mortality, and this was valid for the three tested isolates. The LD_90_ values were similar for the susceptible and the two resistant *A. fumigatus* isolates. It is known that antimicrobial resistance is often associated with lower pathogenicity, but this was not observed for *A*. *fumigatus* in the present study. In accordance with our results, Valsecchi et al. [19] showed that there was no fitness cost for azole resistant *A. fumigatus* strains in immune-suppressed OF1 mice or in vitro compared to the parental strain. Similarly, in patients, it was shown, in an international multicenter prospective study, that azole-resistant *A. fumigatus* isolates had the same level of pathogenicity compared to susceptible isolates [20].

We used the *G. mellonella* aspergillosis model to test the efficacy of antifungals. First, the gold standard antifungal AMB was evaluated for the treatment of infected larvae. The drug administration resulted in a dose-dependent reduction in larvae mortality. Our results are in accordance with previous studies. Indeed, treatment with AMB has been used for the treatment of invasive aspergillosis in *G. mellonella* by several authors, in general as a comparator when testing the therapeutic efficacy of new drugs [21,22]. In one study, larvae treated with AMB at 3 mg/kg had a 90% survival rate, whereas it was <10% for untreated controls [22]. Similarly, Ben Yaakov et al. [21] showed the effectiveness of AMB at 2 mg/kg at improving larval survival compared to a control group.

VRZ was also effective in our model and appeared to be more effective in larvae infected with VRZ-susceptible isolates compared to those infected with the VRZ-resistant isolate. The dose of 1 µg/larva (5 mg/kg) significantly increased the survival of HEGP064 (VRZ-susceptible)-infected larvae (*p* = 0.02) but not the survival of HEGP2666 (VRZ-resistant)-infected ones (*p* = 0.72). By using a high dose of VRZ, namely, four times the dose used in human therapy (8 µg/larvae, 40 mg/kg), treatment significantly reduced the mortality of the infected larvae even for the VRZ-resistant isolate. Nevertheless, the efficacy remained higher for the VRZ-susceptible isolate compared to the VRZ-resistant isolate. These results demonstrate that the *G. mellonella* model can be used to evaluate azole-resistance in vivo.

In accordance with our results, a previous study [23] concluded that median survival time was prolonged by VRZ treatment when larvae were infected by *A. fumigatus* azole-susceptible isolates (*CYP51A* wild type) but not azole-resistant isolates (*CYP51A* with G54W or TR34/L98H mutations). It was also shown that a high dose of VRZ (32 µg/larva) prolonged the survival of infected larvae, even when the infection was of the VRZ-resistant TR34/L98H mutant [23]. In another study, the *G. mellonella* aspergillosis model was also used to test the response to VRZ treatment in larvae infected with strains that had high MICs for VRZ but were not actually resistant (MICs were below the clinical breakpoint of resistance). Even for these strains with moderate increases in MIC, the in vivo efficacy of VRZ (at 2 µg/larva (10 mg/kg)) was lower than for the susceptible control, demonstrating that the *G. mellonella* model is robust enough to detect slight differences in susceptibility [24].

Besides the use of mortality as an endpoint, we also performed histopathological studies that proved useful to confirm the invasive nature of the infection. Moreover, we confirmed that the efficacy of VRZ treatment was associated with fewer and less severe tissue lesions in treated animals compared to untreated controls. These results confirm and extend previous studies that used histopathology in models of invasive fungal infections in *G. mellonella* [25,26]. One limitation of our study is that only one larva was tested for each condition (strain, dose of VRZ and time-point after treatment).

In the present study, pharmacokinetic parameters of VRZ were determined in *A. fumigatus* infected and non-infected larvae. Concentration of the drug showed a peak 30 min after injection then a gradual decrease over the 24 h period. Interestingly, we observed that infection did not significantly alter the pharmacokinetics of the drug. As previously reported for antibacterial [27] and antifungal [23] drugs, we observed clear relationships between the pharmacokinetic parameters and the therapeutic efficacy. Nevertheless, increasing the dose of VRZ up to 8 µg/larva resulted in efficacy even against the VRZ-resistant isolate. This could be attributed to hemolymph VRZ concentrations that were higher than the MIC of the isolate. It has to be noticed that the clinical efficacy and toxicity of the highest dose (16 µg/larva) evaluated in the pharmacokinetic experiments were not tested.

## 5. Conclusions

*Galleria mellonella* is a suitable model of invasive aspergillosis. In contrast with in vitro tests, this easy-to-handle animal model, which is associated with the role of the innate immune response, allows one to reliably assess mortality and to evaluate the in vivo efficacy of azole treatment against both susceptible and resistant isolates. It will be an interesting tool to further explore the correlations between the in vitro activity and in vivo efficacy of antifungal drugs.

## Figures and Tables

**Table 1:** Types of mutations for CYP51, and azoles’ minimal inhibitory concentration values against *Aspergillus fumigatus* isolates HEGP064, HEGP4017 and HEGP2666.

Isolate	CYP51A Mutation	MIC (mg/L)			
		AMB	ITZ	VRZ	PSZ
HEGP064	WT	0.5	0.25	0.5	0.06
HEGP4017	G54W	0.5	>8	0.5	>8
HEGP2666	TR34/L98H	0.5	>8	8	1

WT: wild type, AMB: amphotericin B, ITZ: itraconazole, VRZ: voriconazole, PSZ: posaconazole.

**Table 2:** Lethal dose 90% (LD_90_) and 10% (LD_10_) for *Aspergillus fumigatus* isolates.

Isolate	Susceptibility ITZ/VRZ	LD_10_ (CFU/mL)	LD_90_ (CFU/mL)
HEGP064	S/S	6.01 × 10^5^	4.88 × 10^7^
HEGP4017	R/S	1.18 × 10^6^	9.59 × 10^7^
HEGP2666	R/R	1.24 × 10^6^	1.01 × 10^8^

ITZ: itraconazole, VRZ: voriconazole, R: resistant, S: susceptible, CFU: colony-forming unit.

**Table 3:** Pharmacokinetic profile of voriconazole in the hemolymph of *Galleria mellonella* larvae.

	Pharmacokinetic Parameters of Voriconazole					
Dose ^a^ (µg/Larva)	Uninfected Larvae			Infected Larvae		
	C_max_ (mg/L)	C_min_ (mg/L)	AUC_0–24_ (mg·h/L)	C_max_ (mg/L)	C_min_ (mg/L)	AUC_0–24_ (mg·h/L)
0.5	2.5 ± 0.51	0.11 ± 0.04	5	1.74 ± 0.09	0.12 ± 0.09	16
1	3.8 ± 0.96	0.13 ± 0.06	23	10.04 ± 1.44	0.09 ± 0.01	64
2	5.86 ± 1.30	0.75 ± 0.07	62	6.71 ± 1.21	0.07 ± 0.01	72
4	16.54 ± 2.51	0.46 ± 0.25	163	11.69 ± 2.13	0.21 ± 0.27	139
8	38.08 ± 9.20	0.32 ± 0.21	309	32.41 ± 3.35	9.85 ± 4.38	317
16	80.61 ± 2.09	11.30 ± 1.50	1073	68.99 ± 4.51	28.17 ± 13.02	1144

^a^ The doses of 0.5, 1, 2, 4, 8 and 16 µg/larva are equivalent to 2.5, 5, 10, 20, 40 and 80 mg/kg, respectively. AUC_0–24_: area under the concentration–time curve from time zero to the 24 h endpoint. C_max_: maximum concentration. C_min_: minimum concentration.

## Data Availability

The data presented in this study are available on request from the corresponding author.

## Supplementary Materials

The following are available online at https://www.mdpi.com/article/10.3390/jof7121012/s1, Figure S1: Direct examination showing hyphae with dichotomous branches in an infected larva inoculated with conidia of *Aspergillus fumigatus* isolate HEGP4017 at 10^7^ CFU/mL. Table S1: Classification of stage of lesion observed in histopathological slides of infected *Galleria mellonella* larvae.
