# Supplementary material for: In Vivo Efficacy of Voriconazole in a Galleria mellonella Model of Invasive Infection Due to Azole-Susceptible or Resistant Aspergillus fumigatus Isolates
Source: J Fungi (Basel). 2021 Nov 26;7(12):1012. doi: 10.3390/jof7121012 (PMC8708373; doi:10.3390/jof7121012)
Supplement: Supplementary file 1 [file jof-07-01012-s001.zip › jof-1422248-supplementary.pdf]

Table S1. Classification of the histopathological lesions observed in infected and treated *G.mellonella* larvae.

Stages	Description
Stage 1: Minimal lesions	Low invasion, rare and small foci containing mostly conidia
	Germinated conidia and hyphae
Stage 2: Mild to moderate lesions	2-a Not extensive
	2-b More extensive
Stage 3: Marked lesions	Extensive lesions with invasion and presence of numerous intralesional hyphae

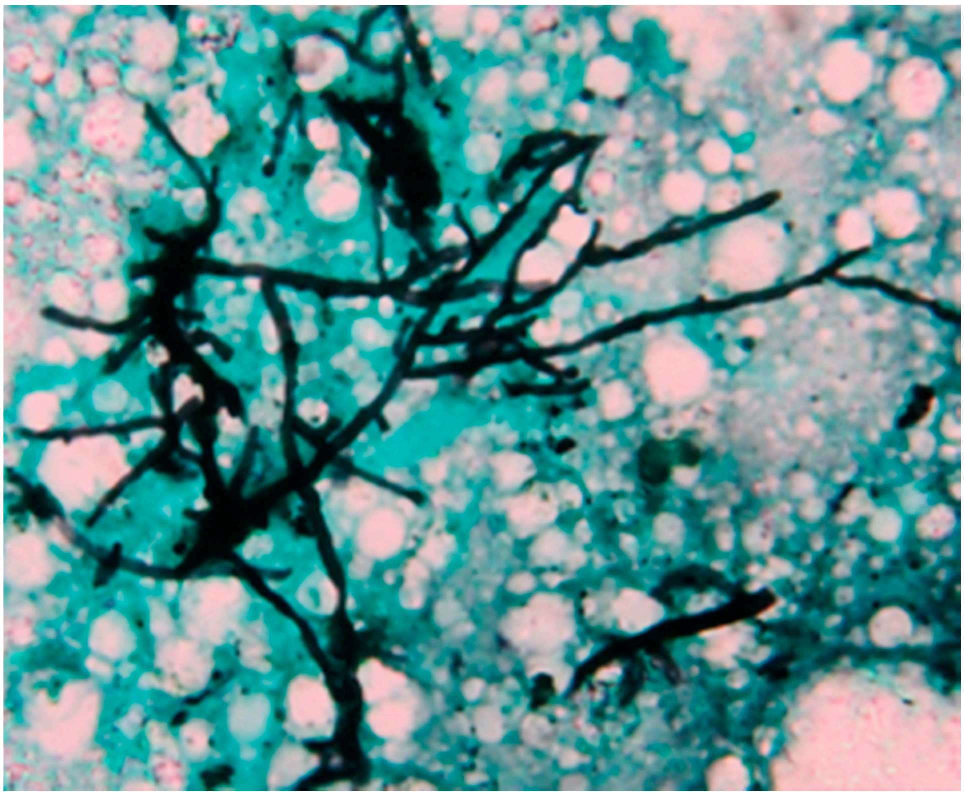

Figure S1. Direct examination of a larva infected with 10^7 cfu/ml of HEGP4017 *Aspergillus fumigatus* isolate. Gomori methenamine silver stain showing proliferating hyphae.
